# Exploring the Usability of a Mobile App for Adolescent Obesity Management

**DOI:** 10.2196/mhealth.3262

**Published:** 2014-06-27

**Authors:** Grace O'Malley, Grainne Dowdall, Amanda Burls, Ivan J Perry, Noirin Curran

**Affiliations:** ^1^Department of PhysiotherapyTemple Street Children’s University HospitalDublinIreland; ^2^Department of Epidemiology and Public HealthUniversity College CorkCorkIreland; ^3^Child Health Information CentreTemple Street Children’s University HospitalDublinIreland; ^4^School of Health SciencesCity University LondonLondonUnited Kingdom; ^5^Department of Applied PsychologyHuman Factors Research GroupUniversity of College CorkCorkIreland

**Keywords:** obesity, mobile health, usability testing, adolescent, participatory health care

## Abstract

**Background:**

Obesity is a global epidemic. Behavioral change approaches towards improving nutrition, increasing physical activity level, improving sleep, and reducing sitting time are recommended as best practices in adolescent obesity management. However, access to evidence-based treatment is limited and portable technologies such as mobile apps may provide a useful platform to deliver such lifestyle interventions. No evidence-based validated app exists for obesity intervention; therefore, a novel mobile app (Reactivate) was developed for use in the Temple Street W82GO Healthy Lifestyles Program (W82GO).

**Objective:**

This study aimed to test the usability (technical effectiveness, efficiency, and user satisfaction) of the Reactivate mobile app in obese adolescents.

**Methods:**

Ten adolescents (7 males and 3 females, aged 12-17 years) who had been treated for obesity (>98th percentile for body mass index) at the Temple Street Children's University Hospital were recruited. Participants were given 8 tasks to complete in order to test the technical effectiveness of the app. A research assistant timed the user while completing each task in order to test the relative user efficiency of the app (time-on-task). The tasks fell into 5 categories and required the user to enter personal settings, find and answer surveys, create a message, use the goal setting feature, and enter details regarding their weight and height. In exploration of user satisfaction, each participant completed the standardized software usability measurement inventory (SUMI), which measures 5 aspects of user satisfaction: efficiency, effect, helpfulness, controllability, and learnability. Descriptive statistics were used to explore the mean relative user efficiency and SUMI scores.

**Results:**

Mean age was 14.26 (SD 1.58) years. All adolescents completed each of the tasks successfully. The mean relative user efficiency scores were two to three times that of an expert user. Users responded that they would use Reactivate to monitor their growth over time, for motivation, and for goal setting. All users described Reactivate as an important mobile app.

**Conclusions:**

Our study describes the usability of a mobile app used in adolescent obesity management. Adolescents found Reactivate easy to use and their SUMI results indicated that the app scored high on user satisfaction. Usability testing is an important step towards refining the development of the Reactivate app, which can be used in the treatment of obesity. The study on the clinical efficacy of the Reactivate app is currently underway.

## Introduction

For adolescents who are identified as being obese, prompt and effective lifestyle interventions are required in order to minimize associated comorbidities and to prevent further progression of obesity into adulthood. Due to cost and resource limitations, effective obesity interventions can be challenging to deliver to the adolescent population in need of care. The Temple Street W82GO Healthy Lifestyles Program, (as of May 2014) is Ireland’s only obesity treatment for children and adolescents [1]. Based on recent data, it is estimated that there are around 100,000 children and adolescents who are clinically obese in Ireland [2]. With current clinical services facilitating the treatment of approximately 150 families per year, it is clear that efforts to scale up treatment are needed.

**Figure 1:**
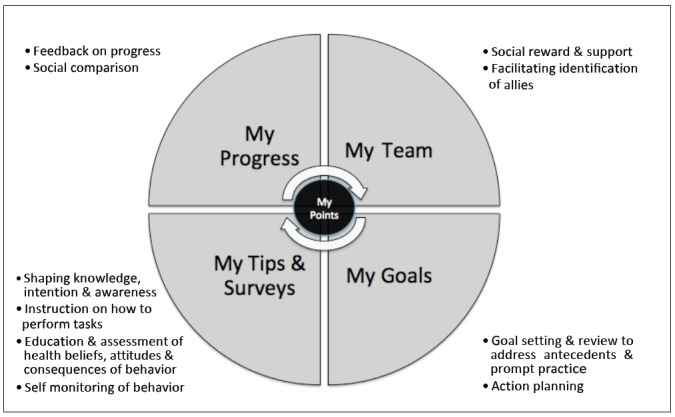
Schematic of Reactivate behavioral change components.

**Figure 2:**
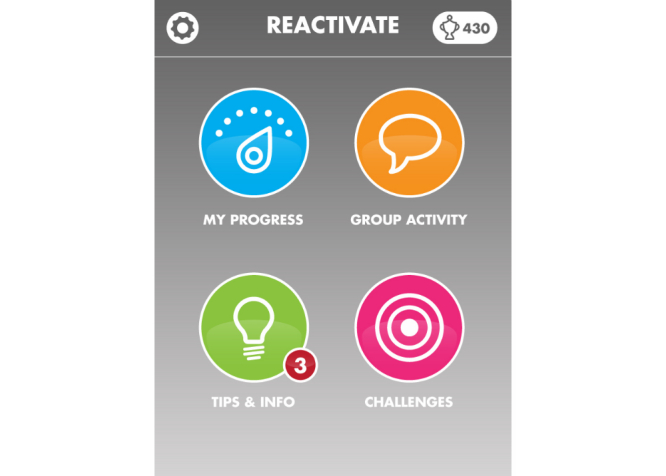
Screenshot of Reactivate home screen.

Given the development of mobile technology, it may be possible to adapt face-to-face obesity interventions for a mobile platform and deliver secure and effective care remotely [3,4]. Previous work in the area of mobile health has highlighted the potential benefits of including a remote treatment option in the management of chronic disease [5-7]. In addition, recent work in adult weight management has suggested that mobile health interventions may be effective [8]. The effective design and development of mobile health interventions is influenced by adequate evaluation of the device interface by the end user, such that an iterative cycle of development can support optimal functioning of the remote device/intervention [9]. Little data exists regarding the use of mobile health interventions in adolescents, although studies have reported that short message service (SMS) texting and image-based interventions are acceptable and perceived as relevant to adolescents who are obese [10,11]. In an effort to augment the W82GO service, the Reactivate mobile app has been designed as a remote treatment aid for adolescents who are obese. Development of the Reactivate app included participation by end users and a previous study examining the acceptability of such a mobile app in a separate cohort of parent-child dyads (unpublished data). In short, semi-structured interviews and two focus groups with service users were undertaken to collect qualitative data regarding the necessity of such an app for obesity treatment and the features it must include. The main features and issues described by participants included design attributes, the perceived benefits of using an app for treatment, concerns regarding data protection, and privacy. Design of the Reactivate app was facilitated by contemporarily published evidence-based studies related to obesity interventions and by results from the acceptability study. In brief, the app is underpinned by the social cognitive theory, the theory of planned behavior, and the capability, opportunity, and motivation (COM-B) framework [12-14]. It incorporates behavioral change tools such as self-monitoring, goal setting, a rewards system, and peer support (Figures 1 and 2). Evidence-based tips such as education regarding the importance of sleep for weight management [15] are sent to the user in the form of a text, video, or an image and the user is encouraged to engage in daily goal setting and goal review.

Although thousands of commercial health and fitness-related mobile apps exist, few developers report whether apps have been developed in line with best-practice guidelines [3,16] or with the end user in mind [17]. The user experience with mobile apps varies depending on the type of mobile used and users often report difficulty using mobile apps [18] due to small screen size, limited processing power, and the incompatibility of apps across devices [19]. It is vital that the end user is considered throughout the app development process (particularly where the app is to be used in clinical cohorts) and that testing for both technical and clinical effectiveness is completed so that functionality can be optimized. Recently, electronic health interventions have been evaluated for usability and their testing has assisted in developing interventions for chronic conditions, which are technically effective and acceptable for use in adolescents [20]. The current study aimed to test the usability of the Reactivate mobile app with a clinical cohort of adolescents who were obese.

## Methods

### Overview

Usability was defined as the extent to which the app could be used by a clinical cohort of obese adolescents, to achieve specified tasks with technical effectiveness, efficiency, and satisfaction. This definition is in line with the international organization of standardization (ISO) 9241-11 [21].

### Participants

Parent-adolescent dyads attending the W82GO healthy lifestyles program at Temple Street Children's University Hospital, Dublin, Ireland for at least six months were invited to participate in the study. Adolescents attending the service had a diagnosis of clinical obesity (body mass index >98^th^ percentile). The study was approved by the ethics committee of Temple Street Children's University Hospital (TSCUH 11-024). Participants were excluded if the adolescent resided in foster care, if they had a moderate to severe learning disability, and/or if either the adolescent or parent were not proficient in understanding English. Adolescents and their parents who agreed to participate signed age-appropriate consents and assents.

### Procedure

Usability testing methods proposed by Kushniruk et al and Schneiderman were followed [22,23]. A test plan of three stages was developed. Stage 1, sought to test the technical effectiveness of the app (ie, whether the user could complete a given task or not). Stage 2, tested the relative user efficiency of the app, with the user being timed while he/she undertook standardized tasks in order to examine whether the app was easy to navigate. Stage 3, examined user satisfaction with the app. Subsequently, participants representative of the end users were recruited and 8 representative tasks using the Reactivate app were chosen for testing. Prior to testing, the Reactivate app was installed on 10 Android mobiles (Samsung Galaxy Y). All devices were fully charged and the Reactivate app was tested to ensure that it had downloaded correctly, was functioning without error, and was connected to the WiFi network.

Finally, the manner by which the testing would take place was planned. A usability testing booklet was developed for participants and for testers in collaboration with the human factors research group at the University College Cork – National University of Ireland, in Cork, Ireland. The usability testing was undertaken at the Vodafone user experience center in Dublin and each adolescent was accompanied by a research assistant/tester. The research assistant and each adolescent participant were advised that the aim was to test the app and not the participant. They received written and verbal information regarding the testing procedure, and study participants also received a brief introduction to the app before usability testing commenced.

### Technical Effectiveness

Participants were given 8 tasks to complete in order to test the technical effectiveness of the app. Each task required, or requested, that the participant obtained or entered specific data that would be used in a typical task (Table 1). The task was completed when the tester indicated that the task goal had been obtained (whether successfully or unsuccessfully) or when the participant requested and received sufficient guidance to warrant scoring the scenario as a critical error.

A critical error was defined as an error resulting in an incorrect or incomplete outcome. If a participant required assistance in order to achieve a correct output, then the task was scored as a critical error and the overall completion rate for the task was affected. A noncritical error was an error that would not have an impact on the final output of the task but resulted in the task being completed less efficiently. These errors could also be associated with confusion (eg, selecting the wrong function initially, or using a user interface control incorrectly such as attempting to edit an non-editable field).

**Table 1:** Testing tasks.

Task	Description
Task 1	Find and answer the mood survey.
Task 2	Enter your personal settings into the app and save them.
Task 3	Look at the Fizzy drink video in the *Tips* section and choose that you 'like' it. Submit your response.
Task 4	Send a message saying what day it is today and what age you are.
Task 5	Go to the *My Goals* section and pick 2 goals - (one goal from the *Chill* section and one from *Change* section) Make these goals for everyday of the week and set a reminder of 6 p.m. for each goal.
Task 6	Go to the *My Goals* section and add this new personal goal to the *Fuel* section – I will try a new type of vegetable today (make this goal for everyday of the week and set the reminder for 4 p.m.)
Task 7	Enter your *height* and *weight* for today, and look at your body mass index (BMI).
Task 8	Look at your BMI for today and post this on the *Message Board*.

### Relative User Efficiency

Relative user efficiency measured the mean time a user took to complete a task in comparison with an expert user of the app [24]. The research assistant timed the user completing each task using a stopwatch in order to test relative user efficiency of the app. Time scores were divided by the time taken by an expert user to complete the task. Throughout all the tasks, the tester kept a written record of any subjective comments made by the adolescent. Upon completion of the tasks, the subjective comments were categorized based on the tasks, time to perform each task, features, and app functionality.

### User Satisfaction

The standardized software usability measurement inventory (SUMI) [1,25] was completed by participants at the end of testing to measure the five aspects of user satisfaction. SUMI follows the ISO 9241, the standard method of testing user satisfaction. SUMI is a reliable and validated standardized questionnaire which uses the agreement type of response. Each questionnaire item takes the format of a statement with a fully anchored 3-point Likert type response, with options being "Agree", "Undecided", and "Disagree". Each item is then scored positively or negatively, depending on the statement, and the scores are summed based on their contribution to each of the five main SUMI factors; efficiency (sense of the degree to which the software enables the task to be completed in a timely, effective, and economical fashion), affect (the respondents emotional feelings towards the software), helpfulness (the perception that the software communicates in a helpful way to assist in the resolution of difficulties), controllability (the feeling that the software responds to user inputs in a consistent way), and learnability (the feeling that it is relatively straightforward to become familiar with the software), and the sixth overall SUMI factor of user satisfaction which gives the global score. A global score of 50 out of 100 is considered to be an average score. Participants completed the SUMI and asked the tester for assistance with wording when necessary. Upon completion of the SUMI, participants were asked to highlight anything they liked about the app or give their suggestions on improving the app. Descriptive statistics of the quantitative data were used to explore the mean relative user efficiency and SUMI scores. Notes and comments recorded during the testing process were also transcribed and emergent themes were grouped together.

## Results

### Participant Characteristics

Twelve adolescents (8 boys and 4 girls, aged between 12 and 17 years) who had been treated for obesity were recruited from the obesity clinic. On the day of testing, two families were unable to attend. Hence, a total of 10 adolescents participated in the study. The mean age of participants was 14.3 (SD 1.6) years old; mean weight was 84.7 (SD 55.9) kilograms; mean height was 164 (SD 11) cm; mean body mass index (BMI) was 31.1 (SD 55.9) m/kg^2^; and BMI standard deviation (SD) score was 2.8 (SD 0.3).

### Technical Effectiveness

All tasks were completed successfully and users commented on how easy the interface was to navigate. Noncritical errors recorded included difficulty in recognizing what the app icons represented (5 participants) and difficulty with reading the text on the app at times (2 participants).

### Relative User Efficiency

The time taken by an expert user to complete each task was 5.93 seconds for task 1; 24.37 seconds for task 2; 8.25 seconds for task 3; 17.37 seconds for task 4; 37.50 seconds for task 5; 16.81 seconds for task 6; 20.82 seconds for task 7 and 16.06 seconds for task 8. The mean relative user efficiency scores (RUS) are detailed in Table 2.

### User Satisfaction

The score results of the SUMI are presented in Table 2. All participants rated the app as being important (n=9) or extremely important (n=1) for them. Comments made by participants throughout testing of the app included the ease of use (n=2); the benefit of the weight tracking and reward systems (n=9), and the appealing look and feel of the app (n=3). Participants commented that improvements were needed so that the app could run on an iPhone (n=1); that the colors could be brighter (n=3), and that the text could be larger (n=2).

**Table 2:** Relative user efficiency and SUMI scores.

Characteristic	Mean (SD)
RUS Task 1 (second)	1.7 (1.3 )
RUS Task 2 (second)	2.4 (1.4)
RUS Task 3 (second)	2.5 (2.1)
RUS Task 4 (second)	1.2 (0.8)
RUS Task 5 (second)	2.2 (1.3)
RUS Task 6 (second)	3.6 (1.7)
RUS Task 7 (second)	2.2 (1.2)
RUS Task 8 (second)	1.7 (1.3)
SUMI Global	64.40 (4.99)
SUMI Efficiency	60.60 (6.70)
SUMI Affect	67.00 (5.06)
SUMI Helpfulness	60.80 (8.63)
SUMI Controllability	60.30 (5.06)
SUMI Learnability	60.80 (9.21)

## Discussion

### Principal Findings

A representative cohort of adolescents who were obese was recruited to test the usability of a mobile app designed for use in the Temple Street W82GO Healthy Lifestyles Program. Adolescents who had already commenced treatment were recruited as it was anticipated that they would already have an understanding regarding the fundamentals of obesity treatment such as planning and goal setting. In addition, we did not exclude participants based on their level of literacy so that the needs of all users could be taken into account. Overall, the results of testing were promising and participants rated the app as important for them and easy to use. Each of the test tasks were completed successfully without critical error indicating that technical effectiveness was achieved.

The relative user efficiency of the app was compared to that of an expert user and the time taken for novice participants to complete tasks was one to three times that of the expert user. As recommended by Bevan [24], measuring the relative user efficiency highlights the potential usability gap between typical users and an expert user and it is anticipated that it often takes normal users two or three times longer to complete a task than an expert. Users were satisfied with the app and reported a number of ways to improve the app further, which were implemented by the developer. A global SUMI score of 64 was promising as 50 is an average score and 68% of software falls within one standard deviation of the mean (ie, scores between 40 and 60) on the SUMI.

To our knowledge, this was the first study to report on the development and usability testing of a mobile app to be used as an adjunct to adolescent obesity intervention. Given the popularity of mobile apps with adolescents and the limited access to evidence-based treatment, we anticipated that a mobile app would be a useful tool for obesity treatment and the results of this study support this. Strengths of the study include the participation of end users in the iterative development process and our use of validated methods for testing.

Few studies have been conducted to assess the usability of mobile app with adolescent patients. One recent study explored the usability of a mobile app for measuring pain in children with cancer [26]. Similar to our findings, participants in the Stinson study [26], commented positively on the aesthetics of the app, on the rewards system, and future use of the app. Testing also revealed important changes to development that were necessary in order for the pain app content to be completely interpreted by adolescents and to avoid navigating away from a chosen page mistakenly. With regard to user satisfaction, participants completed a questionnaire and 86% reported that they liked using the pain app while 79% reported that they found it user-friendly. These results suggest that the app could be used as a tool to assist adolescents in making decisions around pain management. In a similar study involving adult patients with type 2 diabetes mellitus, 25% of participants expressed frustration with using a mobile app as part of their care due to errors in functioning of the app [27] and a systematic review of apps for diabetes management revealed that the look and feel of the app could impact the perceived usefulness of the app [28]. Considering our study against the background of the above study, it is clear that usability testing is paramount for the optimal design and development of mobile apps used in clinical cohorts.

### Limitations

Although the test sample for this study might be considered small in number, a minimum of 8 participants is recommended in heuristic usability testing [29]. We recruited 12 participants for the study but on the day of testing, 2 families could not attend. Given that testing was undertaken with a group of adolescents attending a single urban hospital for weight management, the results cannot be generalized. Future study is warranted to test the usability of the app with a larger number of participants. In addition, the app should be tested in a cohort of adolescents who are not attending a clinic for weight management, as we do not know whether the user’s level of motivation for lifestyle change affects their perceptions regarding technical usability.

In addition, as the testing was undertaken in one building using the same WiFi network, we could not ascertain whether the technical effectiveness could be guaranteed when users are dispersed across the 3G network. However data regarding such limitation will be collected in the ongoing clinical trial. In addition, the clinical trial will reveal whether adolescents engage with the app in a “real-life” scenario over a 12-month period and whether there is a dosing effect with regard to use and effect on health outcome. Finally, we assessed satisfaction of using the app as a whole rather than satisfaction with completing each particular task. Future work to explore each individual component of the app may also be warranted.

### Conclusions

Overall, the Reactivate mobile app performed well in usability testing and the results provide support for its usability by end users. Results of this study guided the final development cycle of the app prior to its use in a randomized controlled clinical trial (NCT01804855). The usability testing of mobile apps designed to address clinical problems is vital, as the needs of the user can be taken into account for better optimization of the mobile app, with respect to its acceptability and utility.

## Abbreviations

BMI: body mass index
HRB: Health Research Board
ISO: International Organization of Standardization
RUS: relative user efficiency score
SUMI: standardized software usability measurement inventory
